# Draft Genome Sequences of Vibrio renopiscarius Strains CECT 8603^T^ and CECT 8604, Two Marine *Gammaproteobacteria* Isolated from Cultured Gilthead Sea Bream (Sparus aurata)

**DOI:** 10.1128/genomeA.00099-15

**Published:** 2015-03-12

**Authors:** David R. Arahal, Lidia Rodrigo-Torres, Teresa Lucena, María J. Pujalte

**Affiliations:** Departamento de Microbiología y Ecología and Colección Española de Cultivos Tipo (CECT), Universitat de València, Valencia, Spain

## Abstract

Vibrio renopiscarius DCR 1-4-2^T^ (CECT 8603^T^) and DCR 1-4-12 (CECT 8604) were isolated from healthy gilthead sea bream (Sparus aurata) from Mediterranean fish farms (Castellón, Spain). Their draft genome sequences (30 and 44 contigs, respectively) have 4.3 Mbp and a G+C content of 45.2 mol% and contain almost 3,700 protein-encoding genes.

## GENOME ANNOUNCEMENT

The genus Vibrio, with more than one hundred species (http://www.bacterio.net/vibrio.html), contains the majority of facultative anaerobic bacteria that can be recovered by cultural methods from the marine environment. Vibrio species have a world-wide distribution, having been isolated from seawater and sediments, marine invertebrates, and fish.

Strains DCR 1-4-2^T^ (CECT 8603^T^) and DCR 1-4-12 (CECT 8304) were isolated from healthy cultured gilthead sea bream (Sparus aurata) from the Mediterranean Spanish coast using marine agar and tryptone soy agar plus 2% NaCl (cultured at 26°C for 48 h). They were presumptively identified as Vibrio ichthyoenteri-like (1), but subsequent characterization permitted us to propose them as a novel species, Vibrio renopiscarius (E. Tarazona, M. A. Ruvira, T. Lucena, M. C. Macián, D. R. Arahal, and M. J. Pujalte, submitted for publication).

V. renopiscarius is a facultative anaerobe; it ferments glucose without gas production and reduces nitrate to nitrite. Cells are coccoid to rod shaped and motile. Oxidase and catalase are positive. This organism grows from 4°C to 28°C and from 2% to 6% NaCl. C_16:1_ *ω*7*c/ω*6*c* is the major fatty acid. It grows on defined medium with several carbohydrates, organic acids, and amino acids as sole carbon and energy sources.

Genome sequencing of strains CECT 8603^T^ and CECT 8304 was done using the Illumina MiSeq system by the Central Service of Support to Experimental Research (SCSIE) of the University of Valencia (Spain). Strain CECT 8603^T^ yielded a total of 3,196,666 reads that were assembled using SeqMan NGen 12.0.1, available as free application in BaseSpace, and a draft genome of 30 contigs more than 500 bp in size was obtained. With the same sequencing strategy, strain CECT 8604 yielded fewer reads (2,666,946) and a few more contigs (44). The draft genome sequences of both strains were quite similar in length (4,339,718 bp and 4,347,351 bp, respectively), with average coverage (109) and G+C content (45.2 mol%).

Automatic gene annotation was carried out by the NCBI Prokaryotic Genomes Annotation Pipeline (PGAP) (http://www.ncbi.nlm.nih.gov/genomes/static/Pipeline.html) (2). The genome of strain CECT 8603^T^ contained 3,684 protein-coding genes, 102 pseudogenes, 25 rRNAs, 103 tRNAs, and 1 noncoding RNA (ncRNA), whereas strain CECT 8604 had 3,690 protein-coding genes, 103 pseudogenes, 28 rRNAs, 108 tRNAs, and 1 ncRNA.

Genome annotation and analysis were also done using the RAST server (http://rast.nmpdr.org/) (3). According to this annotation tool, complete glucolysis/gluconeogenesis, pentose phosphate, Entner-Doudoroff, and glyoxylate pathways, as well as the tricarboxylic acid (TCA) cycle and mixed-acid fermentation, were present in both genomes. Also, a type I restriction-modification system, type IV fimbria apparatus, and several multidrug efflux pumps were detected. The absence of a complete arginine deiminase (ADI) system or acetoin-butanediol synthetizing genes justify the negative response of these strains to arginine dihydrolase (ADH) and Voges-Proskauer (VP) tests.

The coding densities of strains CECT 8603^T^ and CECT 8304 are 85.4% and 85.7%, respectively.

### Nucleotide sequence accession numbers.

These whole-genome shotgun projects have been deposited at DDBJ/ENA/GenBank under the accession numbers JTKH00000000 (strain CECT 8603^T^) and JTKI00000000 (strain CECT 8604). The versions described in this paper are the first version of each.
